# Blindness and cataract in children in developing countries

**Published:** 2009-03

**Authors:** Parikshit Gogate, Mohammad Muhit

**Affiliations:** Head, Department of Paediatric Ophthalmology, Community Eye Care, HV Desai Eye Hospital, Pune 411 028, India. Email: parikshitgogate@hotmail.com; Clinical Research Fellow, International Centre for Eye Health, London School of Hygiene and Tropical Medicine, Keppel Street, London WC1E 7HT, UK.

**8^th^ General Assembly of IAPB**

**Course 2:** Congenital and developmental cataract

**Speakers:** Paul Courtright, Parikshit Gogate, Kuldeep Dole, Mohammad Muhit, Khumbo Kalua, Andrea Zin, Elizabeth Kishiki, Rohit C Khanna

**Session:** Childhood blindness

**Speakers:** Pablo Cibils, Mohammad Muhit, Anna Rius, Deepti Bajaj, Marcela Frazier, M Alamgir Hossain

**Figure:**
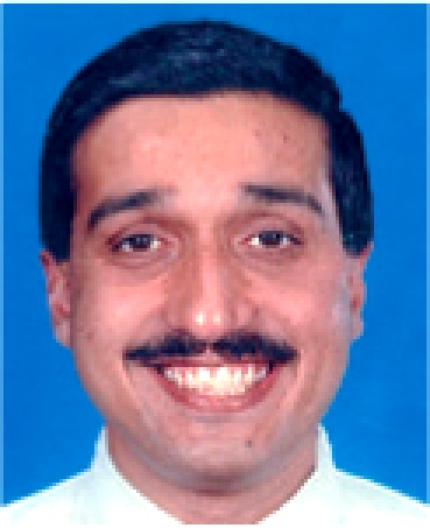


**Figure:**
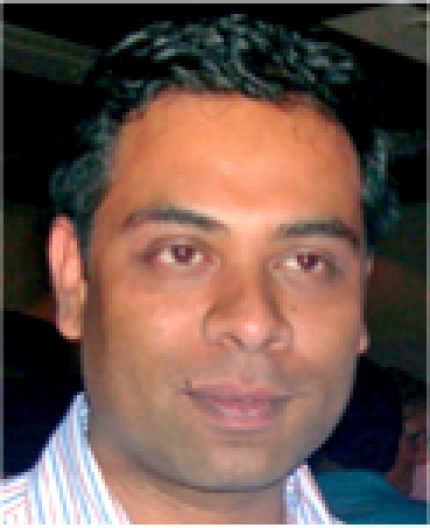


Blindness in children is considered a priority area for VISION 2020, as visually impaired children have a lifetime of blindness ahead of them.^1^ Various studies across the globe show that one-third to half of childhood blindness is either preventable or treatable^1^ and that cataract is the leading treatable cause of blindness in children.^2^

The 8^th^ General Assembly of the International Agency for the Prevention of Blindness (IAPB) provided an opportunity to be acquainted with recent research and programme development work in the prevention of childhood blindness.

## Obtaining population-based data on childhood blindness

Population-based data on childhood blindness are required in order to plan control strategies, but they are difficult to obtain:

- As childhood blindness is ten times rarer than blindness in adults, population-based surveys require a very large sample size.
- Examining blind children in a field study requires special expertise, a trained field team, and special equipment.
- Visual acuity measurement is difficult in very young children and involves special test materials or charts.
- In many communities, blind children are hidden because of stigma.

Methods used for identifying blind children generally target specific locations where children may be found, in order to increase the chances of finding blind children. These methods include examining children in *anganwandis* (kindergartens), schools, vision centres, paediatric eye care centres, and during special outreach initiatives such as *sarva siksha abhiyan* (‘education for all’). The ‘key informant’ method is another means of finding blind children.

### The key informant method

This novel method of obtaining population-based data on childhood blindness has been piloted in Bangladesh, Ghana, Malawi, and Iran^3^^,^^4^^,^^5^^,^^6^

A study in Bangladesh, in which over 75,000 children were screened, compared the key informant and the house-to-house methods. It showed that key informants were able to identify almost two-thirds of all blind children in the study population, and that this required only one-sixth of the time and one-sixth of the human resources, compared to a house-to-house survey. Causes of blindness in children found with both methods were also comparable.

In densely populated Bangladesh, where community network structures are well developed, the key informant approach has shown that there are thousands of children with unoperated cataracts. This approach has also been successful in countries that are less densely populated, such as Ghana and Malawi.^4^^,^^5^

The key informant method provides a way to conduct large-scale population-based studies on childhood blindness in resource-poor countries, in order to obtain valid data on prevalence and causes, which can then be used to plan programmes and policies.

This method is quick, cost-effective, and involves community participation. All the other methods listed above have not proved so useful to detect children with cataract, with the exception of the *sarva siksha abhiyan* scheme, which also uses schoolteachers and health care workers as informants.

**Figure:**
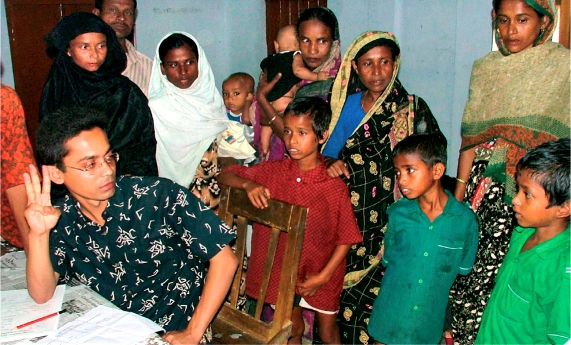
Finding blind children from the community. BANGLADESH

Child Sight Foundation

## Developing programmes to control blindness and cataract in children

Visual impairment in children can have an impact on their performance at school, as well as their social interaction and development. Promoting eye health in children and ensuring early detection of visual impairment is an important part of general eye health and child health strategies.

Since the launch of VISION 2020, various programmes have been developed in resource-poor countries to control blindness and cataract in children. Speakers presented a selection of pilot or established programmes in Latin America and Asia.

### Latin America

Vision screening in children is gaining popularity in many low- and middle-income countries, although there is very limited data available on its effectiveness and impact.

In Brazil, paediatricians have been enlisted to identify leukocoria using the red reflex test. This approach, described by Andrea Zin, has been successful because in this middle-income country most births are institutionalised and almost all children are seen in their infancy by a paediatrician.

Anna Rius presented a pilot project in Nicaragua and El Salvador, which was undertaken to develop and utilise campaign and educational materials in order to train and sensitise schoolteachers and nurses on children's eye health. The project showed that locally developed or adapted training and campaign materials can contribute effectively to a sustainable programme for the promotion of child eye health.

Another pilot study, in Nicaragua, trained teachers and volunteer nurses to screen the vision of schoolchildren. Marcela Frazier presented a study of its long-term outcome. During the pilot project, 5,673 children were screened by nurses and schoolteachers, 376 of whom were then referred to an optometrist; 96 required and received new spectacles. A year later, the follow-up study retraced 51 of those 96 children on a randomly selected day and found that only ten were wearing their spectacles. Further research is needed to determine whether additional education or other factors can improve compliance with spectacle wearing among schoolchildren.

### India

Deepti Bajaj presented the childhood blindness programme in India which was initiated by ORBIS to support the Indian government's goal to create 50 paediatric eye care centres by 2010. Strategies included: creating a child-friendly environment in eye care facilities, training paediatric eye care teams, supplying appropriate technology and essential equipment for paediatric eye care, empowering local communities in case detection, and educating parents on childhood eye diseases and their prevention.

Between 2002 and 2008, ORBIS worked with 24 eye care partners to establish paediatric eye care centres in India, which will have a long-term impact on reducing avoidable blindness in children.

### Bangladesh

MA Hossain described the large-scale programme developed by Sightsavers and ORBIS to control cataract blindness in children in Bangladesh. Since its launch in 2004, a total of 6,562 children with cataract have been identified and 90% of them have received sight-restoring surgery.

## Conclusion

Blindness and cataract in children remain a major challenge in resource-poor countries. Significant work has been done to tackle this problem in terms of research and programme development. We now need to use the expertise and knowledge gained to develop larger and better programmes to achieve the target of VISION 2020, which is to halve the prevalence of childhood blindness and to eliminate cataract blindness in children.

**Surgical management of childhood cataract**

Childhood cataract, congenital or developmental, can be readily treated. However, the surgical management of cataract in children is different from that of adults and the postoperative follow-up takes longer and is more complex.

**Early recognition of childhood cataract**

Early treatment is crucial. Delays in recognition and subsequent surgery can lead to amblyopia later in life. A Tanzanian study showed that there was an 18-month delay between recognition of cataract and surgery.^1^

Barriers to early catatact surgery in children include:

- Lack of awareness amongst parents, especially in rural settings.
- Asymptomatic children who regard their poor vision as ‘normal’.
- Lack of paediatric or anaesthetic services in the region.
- Cost of surgery.
- Lack of awareness amongst general practitioners, paediatricians, and even ophthalmologists, which means that parents may be wrongly advised to ‘wait’ until the child is older.
- Fear of surgery amongst parents, due to concern about the risk of anaesthesia or the experience of poor results in other operated children.
- A belief amongst parents that congenital blindness (including congenital cataract) is simply not treatable.

**Preoperative evaluation**

It should include:

- visual acuity
- intraocular pressure
- keratometry
- biometry (under general anaesthetic if necessary)
- external ocular and fundus examination
- B-scan for opaque media (to rule out ocular morbidity, such as microphthalmos, coloboma, primary persistent hyperplasic vitreous, uveitis, or tumour).

**Surgery**

Paediatric cataract surgery is just one intervention in a series of steps needed to restore the child's vision. The surgery is challenging because the sclera is less rigid and the anterior capsule is elastic. It may be complicated by intraocular haemorrhage or posterior capsule rupture.

- It is widely accepted that cataract extraction with primary intraocular lens (IOL) implantation is safe and effective when performed in children over the age of two by a specialised paediatric surgeon.^*^
- IOLs may be implanted earlier for unilateral cataracts, to reduce the likelihood of amblyopia.
- As the nucleus is invariably soft, the cataract can be aspirated using a Simcoe cannula or an automated irrigation/aspiration probe.
- A primary posterior capsulotomy with anterior vitrectomy is essential up to the age of six and may be necessary in older children if no follow-up is available.
- In-the-bag placement of the IOL is crucial to prevent decentration and capture.
- The wound must always be sutured, even if it is a 2 mm sideport.

**Postoperative care**

Postoperative care and follow-up is extremely important - at least as important as the surgery itself.

- Postoperative inflammation and posterior capsular opacification are very common. Steroid antibiotic drops are required postoperatively for up to two months, in addition to a cycloplegic for the first two weeks. Oral steroids may be required for the first week. An early Nd YAG laser capsulotomy can be performed in older children.
- An accurate refractive correction must be given at first follow-up and amblyopia therapy started in the form of patching the good eye. School-age children need a pupil-split bifocal to take care of their near tasks, but younger children can simply be overcorrected for near.
- Three-monthly review is recommended during the first year and an annual review thereafter.

**Figure:**
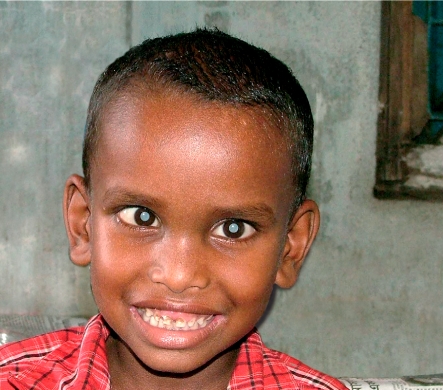
Young boy with cataract blindness. BANGLADESH

Child Sight Foundation

**Counselling parents**

Counselling parents at various stages of care for paediatric cataract should be an integral part of the service.

Parents should be made aware of the importance of postoperative care and of refraction before and after surgery.^2^ Using key informants, not only for detection, but also to encourage and motivate parents to comply with long-term and regular follow-up, has also been very successful in a large-scale programme in Bangladesh.

References1MwendeJBronsardAMoshaMBowmanRGeneauRCourtrightPDelay in presentation to hospital for surgery for congenital and developmental cataract in TanzaniaBr J Ophthalmol200589147814821623445710.1136/bjo.2005.074146PMC17729452EricksenJRBronsardAMoshaMCarmichaelDHallACourtrightPPredictors of poor follow-up in children that had cataract surgeryOphthalmic Epidemiol20061342372431687728210.1080/09286580600672213

## Footnotes

^*^**Note from the Editors** A forthcoming study by the British Isles Congenital Cataract Interest Group (BCCIG) has shown that primary IOL implantation in children under the age of two has been widely adopted among the relatively small number of paediatric ophthalmologists who manage these children in the UK and Ireland. There is concordance in practice with regards to surgical technique, choice of IOL model, and choice of power calculation formula. However, variation exists in eligibility criteria. A national study is now underway through the BCCIG to systematically measure the outcomes and predictors of outcomes of primary IOL implantation in children under the age of two. Lola Solebo A, Russell-Eggitt I, Nischal KN, Moore AT, Rahi JS, on behalf of the British Isles Congenital Cataract Interest Group (BCCIG). Forthcoming paper.
